# Characterization of sexually acquired HIV-1 transmission networks and genetic variation in northern frontier China, 2021–2024

**DOI:** 10.3389/fpubh.2026.1791010

**Published:** 2026-06-08

**Authors:** Yu Liu, Hanping Li, Baobao Bai, Yao Li, Ziwei Liu, Xiaodong Liu, Juan Du, Yongjian Liu, Lei Jia, Jingwan Han, Bohan Zhang, Jingyun Li, Lin Li, Hong Yang

**Affiliations:** 1 School of Public Health, Inner Mongolia Medical University, Hohhot, China; 2 Department of HIV/AIDS Control and Prevention, Inner Mongolia Center for Disease Control and Prevention (Inner Mongolia Academy of Preventive Medicine), Hohhot, China; 3 State Key Laboratory of Pathogen and Biosecurity, Academy of Military Medical Sciences, Beijing, China; 4 Center for STD and AIDS Prevention and Control, Baotou Center for Disease Control and Prevention, Baotou, China; 5 Microbiology Laboratory, Baotou Center for Disease Control and Prevention, Baotou, China

**Keywords:** HIV-1, molecular transmission network, MSM, older heterosexual men, genetic variation, drug resistance

## Abstract

**Background:**

In Baotou, sexual transmission is the primary route of HIV-1 infection among newly reported cases. Given the increasing prevalence in recent years, this study aimed to identify the factors driving local viral spread and analyze the characteristics of pretreatment HIV drug resistance (PDR).

**Methods:**

Plasma samples were collected from 407 newly reported, treatment-naïve individuals infected via sexual transmission in Baotou between 2021 and 2024. HIV-1 pol gene sequences were obtained using next-generation sequencing (NGS) to infer genotypes, phylogenetic relationships, and molecular transmission networks. Logistic regression models identified factors associated with molecular clustering and high network connectivity. PDR was analyzed using the Stanford University HIV Drug Resistance Database.

**Results:**

Of the 407 individuals, 363 (89.2%) yielded valid HIV-1 pol sequences. The predominant subtypes were CRF07_BC (54.3%), CRF01_AE (29.2%), and CRF55_01B (3.8%). Overall, 148 sequences (40.8%) formed 37 molecular transmission clusters, including two large clusters. Centrality indicators revealed that significant differences between older heterosexual men and men who sex with men (MSM) within large transmission clusters. Analysis of drug-resistant transmission networks revealed three small clusters. The prevalence of PDR to any antiretroviral drugs, nucleoside reverse transcriptase inhibitors (NRTIs), non-nucleoside reverse transcriptase inhibitors (NNRTIs), and protease inhibitors (PIs) were 7.99% (29/363), 4.41% (16/363), 2.48% (9/363), and 2.20% (8/363), respectively. NNRTI-associated resistance rates were the highest, with most exhibiting moderate to high resistance levels.

**Conclusion:**

Older heterosexual men maintain active, localized transmission networks in Baotou. Targeted control measures addressing this high-risk subpopulation are essential to complement existing MSM-focused interventions. The prevalence of NNRTI resistance indicates that ongoing surveillance of resistant strains and pretreatment drug resistance testing remain critical for effective clinical management.

## 1 Introduction

During the initial phases of the HIV/AIDS epidemic in China, infection was primarily associated with intravenous drug use (IDU) and unregulated blood collection practices (1, 2). Blood-borne transmission was curtailed, and IDU and vertical transmission decreased, the primary transmission routes gradually shifted to sexual transmission, encompassing both homosexual and heterosexual contact (3). Individuals infected via sexual transmission display distinctive characteristics associated with sex and age (4–7). Among reported male infections, men who have sex with men (MSM) constituted 69.2% of the 15–24 years of age demographic, with this percentage progressively declining to 10.9% in the ≥50 years demographic. In contrast, the percentage of infections attributed to heterosexual transmission rose from 27.6% in the 15–24 age group to 87.4% in the ≥50 age group. Effective control of sexually transmitted HIV/AIDS presents significant short-term challenges. The extensive heterosexual population and diverse sources of infection complicate transmission management. Heterosexual transmission contributes considerably to new infections in China, both presently and in the foreseeable future. Moreover, the distinctive social and sexual practices of MSM render the management of HIV-1 transmission within this cohort a complex global health issue (8, 9). MSM are expected to continue being the demographic with the highest risk of HIV-1 infection.

Situated in the Inner Mongolia Autonomous Region of northern China, Baotou reflects these epidemiological trends. Disclosed statistics indicate that 99.0% of newly reported HIV-1 cases in Baotou are attributed to sexual transmission, a trend projected to persist. The annual incidence of cases reported in Baotou has been rising consistently, ranking among the highest in the 12 cities of Inner Mongolia. We conducted phylogenetic and molecular network analyses on 407 newly reported cases of sexually transmitted infections from 2021 to 2024. This study aimed to enhance our understanding of HIV-1 genetic variation and molecular transmission dynamics, to identify risk factors associated with molecular clustering and high connectivity, and to analyze pretreatment drug resistance (PDR) characteristics. The findings of this study aim to inform targeted prevention and control strategies for sexually transmitted HIV-1-infected populations in Baotou, facilitating more precise public health interventions. Moreover, this study serves as a valuable reference for efforts aimed at the prevention and control of HIV/AIDS throughout the Inner Mongolia Autonomous Region.

## 2 Materials and methods

### 2.1 Study population and data collection

Plasma samples were collected from 407 HIV-infected individuals in Baotou between January 1, 2021, and December 30, 2024. The study participants covered all newly reported infection cases across all banners, counties, and districts in Baotou, with a sample collection rate of 86.96% (407/468). Plasma was separated within 6 h of collection and stored at −80 °C. Inclusion criteria were: (1) newly reported HIV-1 infection; (2) no prior antiretroviral therapy (ART); and (3) infection via sexual contact. Epidemiological information (including age, sex, reported mode of transmission, and baseline CD4 + cell count) was obtained. All samples underwent LAg-avidity EIA, with those exhibiting ODn values <1.5 classified as new cases. The study was approved by the Biomedical Ethics Review Committee of Inner Mongolia Center for Disease Control and Prevention (202411282). Written informed consent was obtained from all participants before sample collection.

### 2.2 HIV-1 genotyping and sequence processing

RNA was extracted from plasma samples collected before the initiation of antiretroviral therapy. To obtain amplicons suitable for next-generation sequencing (NGS), the HIV-1 *pol* gene region covering the PR and first 226 codons of the RT was amplified in triple fragments with one-step RT-PCR (Takara, RR055A) and nested PCR (Takara, RR901A). PCR primers in the Supplementary Table 1. Amplified products were pooled according to the DNA quantification results, ensuring that the DNA quantity per sample was at least 50 ng and the total library DNA content was at least 150 ng. Amplicon fragments were sequenced on the Illumina platform using sequencing-by-synthesis (10). A 20% consensus threshold was used to generate sequences in clustering analyses. This threshold ensures high consistency between NGS and standard Sanger sequencing. Sequence quality was assessed using quality control protocols, aligned with MAFFT, and manually edited. Subtypes were inferred using the BLAST and COMET tools and confirmed via maximum-likelihood phylogeny with 1,000 ultrafast bootstrap replicates. All 363 pol sequences generated in this study have been submitted to the GenBank database (accession numbers: PZ014999-PZ015361).

### 2.3 Phylogenetic reconstruction

Maximum-likelihood (ML) phylogenetic trees were constructed using FastTree 2.1.10, employing a GTR + CAT nucleotide substitution model, SPR = 4, and the Shimodaira-Hasegawa test to estimate node support. Reference sequences representing major global and Chinese circulating recombinant forms (CRFs) were included. Clusters were visually inspected using FigTree.

### 2.4 Molecular network construction

HyPhy 2.2.4 was used to calculate the genetic distance between sequences using the Tamura-Nei 93 model. Calculate the number of nodes and clusters across a range of thresholds from 0.001 to 0.020. Select the threshold yielding the maximum number of nodes to further construct the molecular network. The threshold for network construction was set at 0.013 substitutions per site. Transmission networks were visualized using Cytoscape 3.6.0 and Chiplot^1^. Large transmission clusters were defined using a threshold of 10% of the total network nodes. Nodes with a network degree greater than 5 were defined as high linkage nodes.

### 2.5 Pretreatment drug resistance analysis

According to the National Technical Specifications for AIDS Testing, as outlined by the Chinese Center for Disease Control and Prevention, the sequenced data were forwarded to the HIV Drug Resistance Database of Stanford University. The clinical significance of drug resistance is categorized into five distinct levels for each antiretroviral medication: susceptible, possible resistance, low resistance, moderate resistance, and high resistance. Antiretroviral analysis in this study including non-nucleotide reverse transcriptase inhibitors (NNRTIs, doravirine (DOR), efavirenz (EFV), etravirine (ETR), nevirapine (NVP), rilpivirine (RPV), darunavir (DRV)), nucleotide reverse transcriptase inhibitors (NRTIs, abacavir (ABC), zidovudine (AZT), stavudine (D4T), didanosine (DDI), emtricitabine (FTC), lamivudine (3TC), tenofovir (TDF)) and proteinase inhibitors (PIs, atazanavir/r (ATV/r), darunavir/r (DRV/r), lopinavir/r (LPV/r), fosamprenavir/r (FPV/r), indinavir/r (IDV/r), nelfinavir (NFV), saquinavir/r (SQV/r), tipranavir/r (TPV/r)). Any level of resistance, from low to high, is deemed as drug resistant.

### 2.6 Statistical analysis

Demographic data are presented using frequencies and percentages. Descriptive statistics for continuous variables are reported as median and interquartile range (IQR). Univariate and multivariate logistic regression analyses identified demographic and clinical variables associated with molecular clustering and high connectivity: group assignment, viral subtype, CD4 + cell count, and marital status. The difference between older heterosexual men and MSM was determined by using Kruskal-Wallis *H* test. A stepwise regression approach was used to construct the final model, retaining only variables that remained statistically significant (*p* < 0.05) after controlling for other factors. All *p*-values reflect two-tailed tests. Data processing was performed using IBM SPSS 25, and figures were produced using GraphPad Prism 8 and R v4.4.2.

## 3 Results

### 3.1 Study population

Among the 407 cases, 363 (89.19%, 363/407) yielded valid sequences, 44 samples were excluded due to sequencing failure. The median patient age was 37 years. The majority were men (93.94%, 341/363), and MSM accounted for 66.67% (242/363) of the infections, followed by heterosexual men (27.27%, 99/363). Among MSM and heterosexual men (<50 years), a higher proportion were single (64.46%, 156/242; 62.26%,33/53), whereas older heterosexual individuals were predominantly married (69.57%, 32/46) (Table 1).

**Table 1:** Socio-demographic characteristics of HIV-1 infected in different groups in Baotou.

Variable	Total number (*N* = 363)		Heterosexual men (age < 50) (*n* = 53)		Heterosexual men (age ≥ 50) (*n* = 46)		MSM (*n* = 242)		Female (*n* = 22)		χ^2^	*p*-value
	*n*	%	*n*	%	*n*	%	*n*	%	*n*	%		
Year											19.918	0.018
2021	80	22.04	12	22.64	6	13.04	57	23.55	5	22.73		
2022	87	23.97	9	16.98	11	23.91	63	26.03	4	18.18		
2023	98	27.00	9	16.98	12	26.09	73	30.17	4	18.18		
2024	98	27.00	23	43.39	17	36.96	49	20.25	9	40.91		
Age											154.461	<0.001
<50	261	71.91	53	100	0	0	197	81.40	11	50.00		
≥50	102	28.10	0	0	46	100	45	18.56	11	50.00		
Marital status											77.378	<0.001
Single	196	54.00	33	62.26	3	6.52	156	64.46	4	18.18		
Married	106	29.20	11	20.76	32	69.57	48	19.84	15	68.18		
Divorced/Widowed	61	16.84	9	16.98	11	23.91	38	15.70	3	13.64		
Subtype											6.180	0.722
CRF07_BC	197	54.27	30	56.60	31	67.39	123	50.83	13	59.09		
CRF01_AE	106	29.20	17	32.08	9	19.57	75	30.99	5	22.73		
CRF55_01B	22	6.06	2	3.77	2	4.35	17	7.02	1	4.55		
others	38	10.47	4	7.55	4	8.70	27	11.16	3	13.64		
CD4 + cell count											7.623	0.267
<200	88	24.24	11	20.76	12	26.09	58	23.97	7	31.82		
200–500	181	49.86	22	41.51	24	52.17	122	50.41	13	59.09		
>500	94	25.90	20	37.74	10	21.74	62	25.62	2	9.09		

### 3.2 HIV-1 subtype distribution

**Figure 1:**
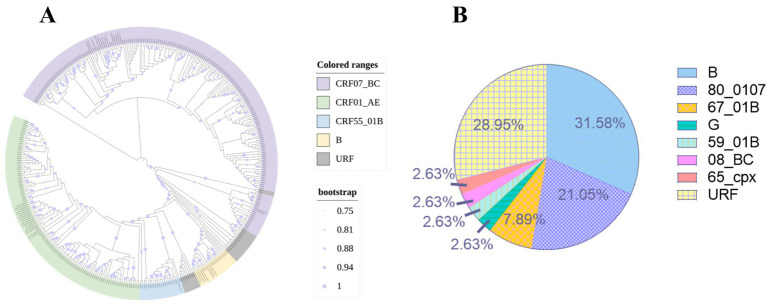
**(A)** Maximum likelihood phylogenetic tree of HIV-1 infections with different subtypes in Baotou City from 2021 to 2024. Branches of different colors represent distinct HIV-1 subtypes and subclades. **(B)** Subtype percentages of other sequences (*n* = 38).

Phylogenetic analysis revealed a diverse landscape comprising more than 10 distinct HIV-1 subtypes in Baotou (Figure 1). The predominant subtypes identified were CRF07_BC (54.27%, 197/363), CRF01_AE (29.20%, 106/363), and CRF55_01B (6.06%, 22/363). Additionally, 38 patients were infected with other subtypes, such as subtype B (*n* = 12), CRF80_0107 (*n* = 8), CRF67_01B (*n* = 3), subtype G (*n* = 1), CRF59_01B (*n* = 1), CRF08_BC (*n* = 1), CRF65_cpx (*n* = 1), and unique recombinant forms (URFs) (*n* = 11). CRF07_BC was the predominant subtype across all three demographic groups, followed by CRF01_AE.

### 3.3 Pretreatment drug resistance

Analysis of drug resistance revealed 29 cases with drug resistance, yielding an overall PDR prevalence of 7.99% (29/363). The prevalence of PDR to NNRTIs, NRTIs, and PIs were 4.41% (16/363), 2.48% (9/363), and 2.20% (8/363), respectively. All PI-associated resistance was categorized as low-level. Among nucleoside NRTI-associated mutations, the most common included M41L + K70T (0.55%, 2/363) and D67E + K70N (0.55%, 2/363), which confer resistance to multiple first-line NRTIs. NNRTI-associated DRMs showed the highest detection rates, with key mutations including K103N (1.38%, 5/363) and E138G (0.55%, 2/363). These mutations primarily confer moderate to high resistance to EFV and NVP (Supplementary Table 1).

### 3.4 HIV-1 transmission clusters

**Figure 2:**
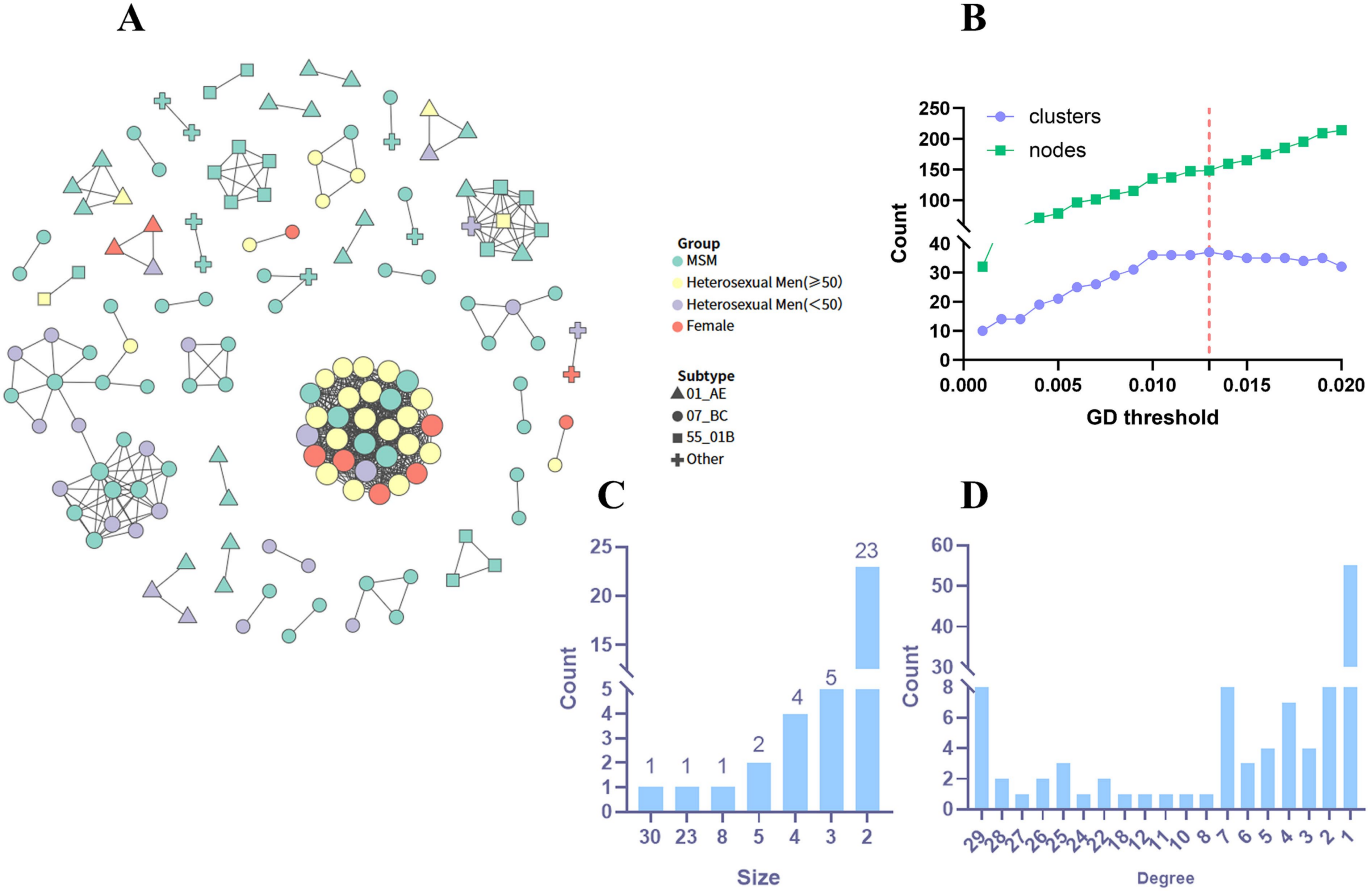
Constructing molecular networks based on a genetic distance of 0.013. Nodes indicate HIV patients. **(A)** HIV-1 transmission network among sexually transmitted populations in Baotou. **(B)** Number of clusters and number of incoming nodes at different genetic distance thresholds. **(C)** Cluster size distribution of the HIV-1 molecular network. **(D)** Degree distribution of the nodes in the molecular network.

**Figure 3:**
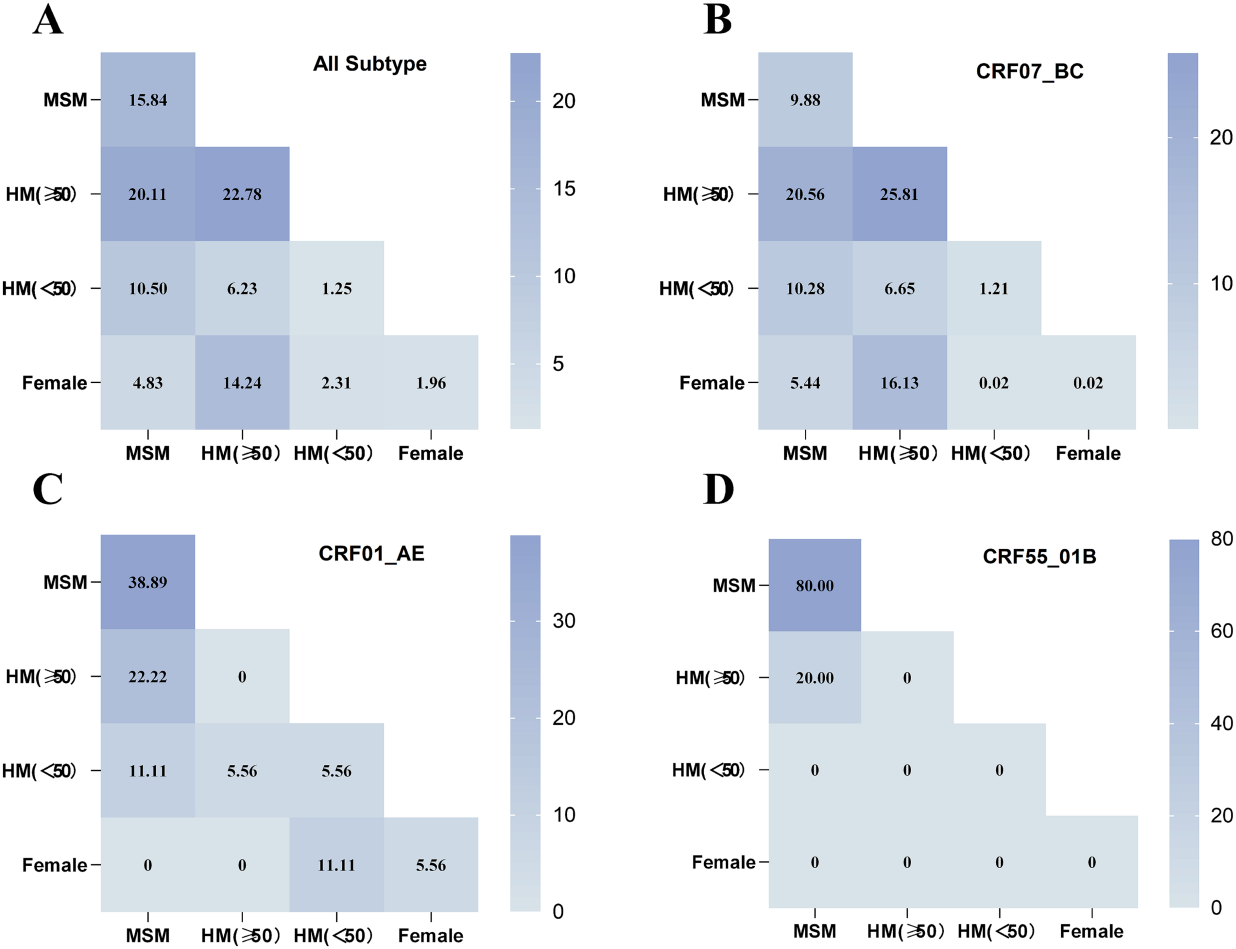
The strength of connections between different risk groups. HM denotes heterosexual men. **(A)** All subtypes (*n* = 562). **(B)** CRF07_BC(*N* = 496). **(C)** CRF01_AE (*n* = 18). **(D)** CRF55_01B (*n* = 25).

Select the threshold corresponding to the maximum number of nodes to further construct the molecular network. A total of 148 sequences formed 37 transmission clusters (40.77%, 148/363) (Figure 2). Among the clustered individuals, CRF07_BC was the predominant subtype (64.86%, 96/148), followed by CRF01_AE (16.89%, 25/148) and CRF55_01B (11.49%, 17/148). MSM constituted the majority of the clusters (59.46%, 88/148), while heterosexual transmission was notably present among men aged ≥50 years (18.24%, 27/148) and those <50 years (15.54%, 23/148). Analysis of marital status showed that 47.97% (71/148) of clustered participants were single, while 41.22% (61/148) and 10.81% (16/148) were married or divorced/widowed, respectively. Network analysis revealed node degrees ranging from 1 to 29. Compared to other groups, transmission among older heterosexual men predominantly involved the CRF07_BC strain. The CRF01_AE strain was more frequently detected in transmission events within MSM populations (Figure 3).

### 3.5 Factors associated with clustering and high linkage

**Figure 4:**
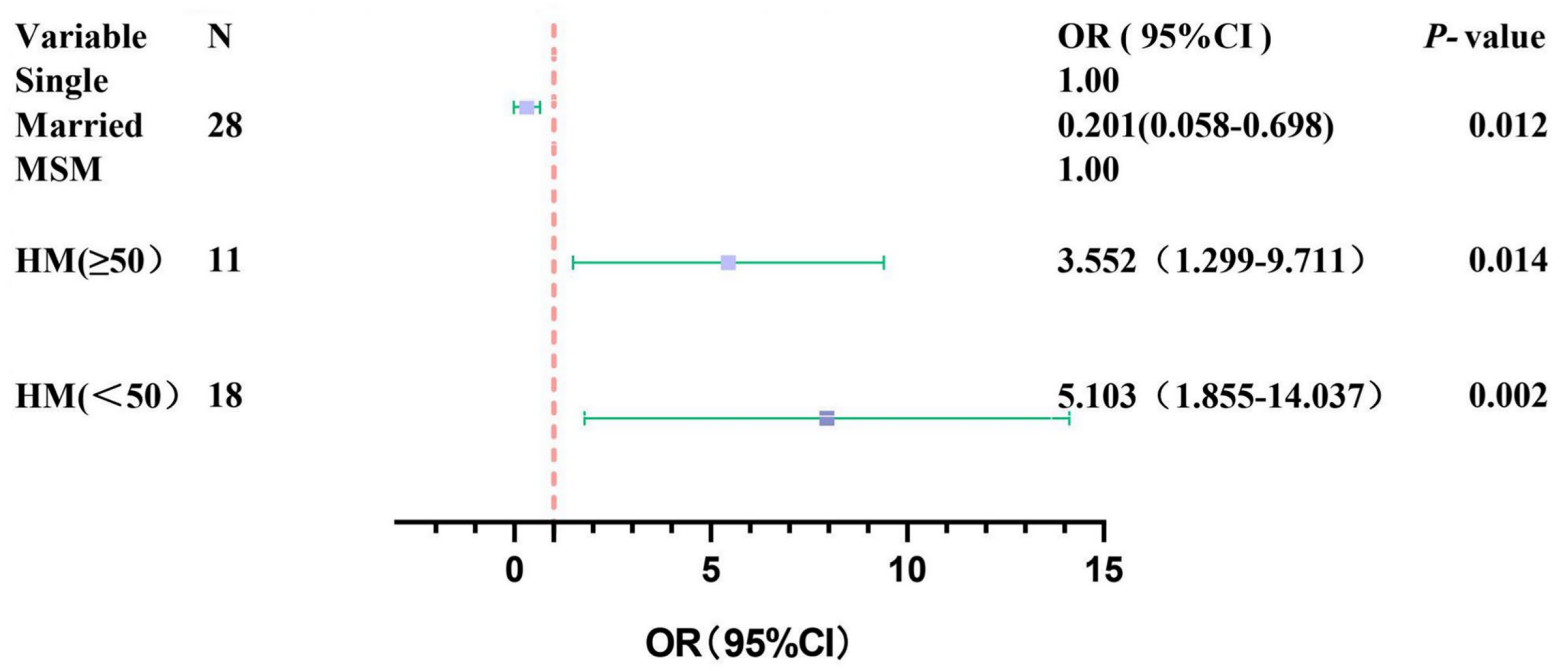
Predictors of large transmission clusters in demographic studies. HM denotes heterosexual men. MSM served as the comparison group for all categories, and single individuals served as the comparison group for marital status.

Multivariate analysis indicated that individuals infected with CRF07_BC (aOR = 2.84, 95% CI: 1.65–4.87) and CRF55_01B (aOR = 8.72, 95% CI: 2.84–26.78) had a significantly higher likelihood of clustering than those infected with CRF01_AE. Additionally, married individuals were more likely to form transmission clusters than unmarried individuals (aOR = 1.90, 95% CI: 1.08–3.36) (Table 2). Compared to MSM populations, heterosexual individuals living with HIV were significantly associated with high linkage (aOR = 3.55, 95% CI: 1.30–9.71; aOR = 5.10, 95% CI: 1.86–14.04, respectively) (Figure 4).

**Table 2:** Analysis of risk factors for HIV-1 infection clusters among sexually transmitted individuals in Baotou.

Variables	Clustered (%)	*p*-value	Crude OR (95% CI)	*p*-value	Adjusted OR (95% CI)
Subtype					
CRF01_AE	25 (23.58)		1		1
CRF07_BC	96 (48.73)	**<0.001**	**3.080 (1.816–5.223)**	**<0.001**	**2.836 (1.651–4.871)**
CRF55_01B	17 (77.27)	**<0.001**	**10.368 (3.452–31.140)**	**<0.001**	**8.723 (2.841–26.781)**
Other	10 (26.32)	0.568	1.273 (0.555–2.917)	0.718	1.170 (0.499–2.740)
Group					
MSM	88 (36.36)		1		1
Heterosexual men (≥50)	27 (58.70)	**0.005**	**2.487 (1.308–4.729)**	0.114	1.831 (0.865–3.879)
Heterosexual men (<50)	23 (43.40)	0.339	1.342 (0.734–2.452)	0.312	1.389 (0.735–2.627)
Female	10 (45.45)	0.400	1.458 (0.605–3.513)	0.966	1.022 (0.379–2.752)
Marital status					
Unmarried	71 (36.22)		1		1
Married	61 (57.55)	**<0.001**	**2.387 (1.472–3.868)**	**0.027**	**1.903 (1.077–3.363)**
Divorced/Widow	16 (26.23)	0.152	0.626 (0.330–1.188)	0.157	0.611 (0.309–1.208)
CD4^+^T lymphocytes (cell/mm^3^)					
<200	30 (34.09)		1		
200–500	36 (19.89)	0.081	1.601 (0.943–2.718)		
>500	82 (87.23)	0.620	1.167 (0.635–2.144)		

Bold values indicates that the variable is statistically significant (*p* <0.05).

### 3.6 Large transmission clusters

Using 10% of the network nodes as the large transmission clusters threshold, two large clusters (LC1 and LC2) were identified, both of which were attributed to CRF07_BC. LC1 comprised 30 highly connected nodes. Notably, 56.67% (17/30) of participants were older men infected through heterosexual contact. LC2 comprised 23 nodes, predominantly MSM (56.52%, 17/23) and younger heterosexual men. Results indicate that the average shortest path length among older heterosexual men (1.00; IQR = 1.00–1.15) was significantly shorter than that among MSM (2.59, IQR1.00–2.91; *p* = 0.006). Additionally, closeness centrality (1.00, IQR = 0.87–1.00 vs. 0.39, IQR = 0.34–1.00; *p* = 0.021) and degree (29.00, IQR = 24.75–29.00 vs. 8.00, IQR = 3.00–29.00; *p* = 0.024) were significantly higher among older heterosexual men (Tables 3, 4).

**Table 3:** Analysis of centrality indicators for different risk groups within large transmission clusters.

Indicators	MSM	Heterosexual men (≥50)	Heterosexual men (<50)	Female	*p*-value
Average shortest path length	2.5909 (1.0000–2.9091)	1.0000 (1.0000–1.1467)	2.8182 (2.0909–3.0909)	1.0689 (1.0000–1.1379)	**0.002**
Betweenness centrality	0.0032 (0.0000–0.0243)	0.0032 (0.0003–0.0032)	0.0021 (0.0000–0.3241)	0.0010 (0.0000–0.0032)	0.522
Closeness centrality	0.3859 (0.3437–1.0000)	1.0000 (0.8723–1.0000)	0.3548 (0.3235–0.4783)	0.9355 (0.8788–1.0000)	**0.002**
Degree	8.0000 (3.0000–29.0000)	29.0000 (24.7500–29.0000)	5.0000 (3.0000–7.0000)	27.0000 (25.0000–29.0000)	**0.001**

Bold values indicates that the variable is statistically significant (*p* <0.05).

**Table 4:** Paired comparison test of different risk groups.

Risk groups^a^	Average shortest path length^b^	Closeness centrality^b^	Degree^b^
HM (≥50) - Female	1.000	1.000	1.000
HM (≥50) - HM (<50)	**0.021**	**0.006**	**0.004**
HM (≥50) - MSM	**0.006**	**0.021**	**0.024**
Female - MSM	**0.035**	0.351	**0.384**
Female - HM (<50)	0.129	0.129	0.103
MSM - HM (<50)	1.000	1.000	1.000

^a^HM denotes Heterosexual Men.

^b^*p*-value adjusted for significance level using the Bonferroni method. Bold values indicates that the variable is statistically significant (*p* <0.05).

### 3.7 Transmission of HIV-1 drug resistance strains

Molecular network analysis identified multiple nodes carrying drug resistance mutations (DRMs) distributed throughout the transmission network (Supplementary Figure 1). The most resistant nodes were located at the periphery of the network and had low degrees. Strains harbored NNRTI-associated mutations, including K103N, K101E, G190A, and K70T, and PI-associated mutations (M46I, Q58E). These resistant strains primarily appeared as isolated nodes or limited transmission pairs, showing no signs of large-scale cluster expansion. Supplementary Figure 1 showed that no drug-resistant sites were detected in LC1, while one sample in LC2 was found to carry the F227L drug-resistant site.

## 4 Discussion

While young MSM represent the majority of HIV-1 infections in Baotou, the largest and most densely linked transmission clusters primarily consist of older, heterosexually infected men. These findings uncover a hidden but active heterosexual transmission network within an epidemic predominantly dominated by MSM. This observation aligns with the distinct demographic-specific patterns of HIV-1 transmission recently reported across China. Ultimately, these data provide a framework for evaluating local epidemic dynamics and designing targeted prevention efforts.

Baotou, as a pivotal transportation and economic center in northern China, may have facilitated increased viral diversity, as indicated by the identification of more than a dozen subtypes. Consistent with national trends, CRF07_BC and CRF01_AE remained the dominant strains, aligning with the major circulating subtypes in China (11). However, the proportion of CRF55_01B—which is primarily prevalent among MSM populations—has been increasing rapidly, becoming the third most common subtype in Baotou and exhibiting distinctive characteristics. Gansu Province and Ningxia Hui Autonomous Region show similar patterns (12). While CRF01_AE primarily impacts heterosexual populations, studies indicate its widespread transmission across geographic regions and high-risk groups, especially within the Chinese MSM population (13, 14). Notably, Baotou’s third-most prevalent subtype, CRF55_01B, originated among MSM in Shenzhen. This subtype has rapidly spread within Chinese MSM populations and further recombined with CRF07_BC to form a third-generation recombinant strain (15, 16). The prevalence of CRF55_01B in Baotou indicates that this subtype has completed cross-regional transmission.

Multivariate logistic regression analysis indicates that viral subtypes and marital status are pivotal factors driving clustering in HIV-1 transmission networks, and that CRF07_BC and CRF55_01B exhibit significantly higher clustering tendencies, potentially reflecting a synergy between viral genetic fitness and host behavioral networks. Previous studies have confirmed that, based on the growth rates values of HIV-1 transmission clusters, CRF07_BC and CRF55_01B exhibit stronger transmission capability than CRF01_AE and subtype B. The unique 7-amino-acid deletion mutation (p6 Δ7) in the p6 Gag protein of CRF07_BC reduces viral infectivity and replication capacity while enhancing transmission efficiency (17). Consequently, this subtype strain has gained a dominant position within highly interconnected sexual networks. In this study, 77.27% of CRF55_01B strains entered the network. As an earlier-emerging recombinant strain, its faster viral load increase and relatively slower CD4 T-cell depletion may help explain the surge and persistent expansion of CRF55_01B among MSM in China (18). In Baotou, where MSM constitute the primary HIV-1 epidemic population, CRF07_BC and CRF55_01B—two subtypes exhibiting specific adaptation to MSM transmission networks—demonstrate synergistic effects between their distinct virological characteristics and the structural features of local high-risk behavioral networks. This synergy significantly enhances their transmission efficiency and clustering.

Previous studies have confirmed that MSM who do not disclose their sexual orientation are more likely to be married. More importantly, this group plays a crucial “bridge” role in HIV transmission networks. Undisclosed MSM exhibit significantly higher closeness centrality than disclosed MSM, with shorter average shortest path length (19). Consequently, they can transmit the virus to others in the network via shorter pathways and at faster speeds, contributing more significantly to viral spread. They transmit the virus within the network through covert sexual activities with male partners, while simultaneously spreading it from high-risk groups to the general population through heterosexual sexual activities with their wives. This creates larger, cross-population transmission clusters.

Compared to other risk populations, MSM exhibit the highest absolute number entering the network, indicating they are the core population locally and drive the most widespread transmission. However, MSM exhibit the lowest proportion of network entry (36.36%, 88/242). Social stigma and identity concerns may reduce MSM willingness to participate in surveillance programs and delay diagnosis (20). Since molecular transmission network construction heavily relies on sampling completeness, undiagnosed or unsorted cases may limit the identification of genetically linked individuals, potentially underestimating the clustering proportion within this risk group. In contrast, older heterosexual men exhibited a higher clustering rate (52.17%, 27/46). This likely reflects differences in testing pathways and transmission structures. Older heterosexual men are more likely to be diagnosed (through public health screenings and medical visits), increasing the probability that epidemiologically linked cases are detected and included in analyses within similar time windows (21). Furthermore, heterosexual transmission within this group often involves commercial sex or spousal transmission within relatively stable, geographically confined networks, potentially resulting in shorter genetic distances and more compact phylogenetic clustering. Despite lower clustering rates, MSM remain the primary drivers of local epidemics due to their absolute contribution to network connectivity. These findings emphasize the need to enhance HIV testing coverage among MSM, reduce stigma barriers, and improve sequencing completeness to enable more precise transmission network reconstruction and more effective intervention strategies.

We observed that heterosexual transmission networks among older men frequently formed high linkage, highlighting a critical blind spot in local HIV prevention strategies. Low HIV awareness, limited testing access, and a reduced willingness to seek medical care due to stigma or comorbidities collectively delay diagnosis and treatment among older adults (22–24). Enhancing the availability of voluntary HIV testing for individuals aged 50 and older, incorporating HIV screening into standard geriatric and primary healthcare services, and offering counseling tailored to age-specific needs are essential strategies for reducing transmission risks. Epidemiological studies indicate that bisexual behavior among married individuals may facilitate bridging transmission. Furthermore, high counts of sexual partners and social stigma within this group contribute to the persistence of high-risk behaviors and delayed detection (25, 26). These factors may help explain why married individuals are more frequently identified within networks and exhibit higher connectivity. The integration of molecular network surveillance with behavioral and clinical data provides a robust mechanism for early identification of clusters and targeted interventions.

We found two large transmission clusters, both of which were dominated by CRF07_BC, validating the high transmissibility and clustering tendency of this CRF07_BC subtype (27). Although MSM dominated the overall case count, the largest active cluster (LC1) was driven by older heterosexual men. LC2 exhibited a more typical transmission pattern primarily driven by MSM. MSM dominate this cluster (56.5%), alongside a significant proportion of younger heterosexual men (39.1%). This cluster exhibits sustained activity levels. Its mixed composition of MSM and young heterosexual men suggests a potential bridging risk, where the virus may spread from the MSM core group to broader heterosexual populations. This underscores the need to strengthen comprehensive interventions targeting MSM while simultaneously addressing transmission risks in other groups (8, 28). In addition, we used four key centrality indicators—average shortest path length, betweenness centrality, closeness centrality, and degree—to examine the position and transmission contribution of different risk groups within large transmission clusters. We focused on and compared two populations: older heterosexual men and MSM. Results for average shortest path length, closeness centrality, and degree showed significant differences between older heterosexual men and MSM, indicating that older heterosexual men were in a central position of large transmission clusters and played an important role in HIV-1 transmission. Older heterosexual men are becoming a key group sustaining localized transmission in the region. Similar age-related clustering patterns have been documented in provinces such as Zhejiang, Sichuan, and Guangxi, where older men sustain localized heterosexual transmission networks contributing to ongoing spread (29–32). These findings mandate a strategic paradigm shift from traditional “key population” targeting to a focus on “high-risk networks.” Current interventions likely prioritize younger MSM cohorts, inadvertently neglecting the escalating risk among older adults. Public health authorities in Baotou must incorporate HIV screening into standard geriatric protocols and chronic disease management to enhance early case detection. Additionally, targeted knowledge education and contact tracing are vital to disrupting the dense transmission chains observed among married older males.

In our study, the overall PDR rate among sexually Acquired HIV-1 infections in Baotou was 9.77%. According to the World Health Organization classification criteria, this placed Baotou within the moderate PDR prevalence category, higher than national average in China (7.8%) (33, 34). Common drug resistance-associated mutations identified in this study include K103N, Q58E, L100I, K101E, K103H, G190A, D67E, K70N/T, and M41L. K103N is the most prevalent NNRTI-associated resistance mutation, conferring high-level resistance to NVP and EFV (35). Q58E is the most common PI-associated resistance mutation, leading to low-level resistance to TPV/r (36). 3TC, EFV, and TDF are widely used and provided free of charge as components of China’s first-line antiretroviral therapy (ART) regimen. The current treatment regimen for infected individuals in Baotou remains primarily based on NRTIs and NNRTIs. Drug resistant cases also exhibited resistance to these drugs in this study. Therefore, prioritizing first-line regimens based on integrase strand transfer inhibitors (INSTIs) can alleviate the impact of NNRTIs and NRTIs resistance (37). Ongoing molecular surveillance remains crucial for tracking the spread of drug-resistant viral strains and developing personalized treatment strategies.

By integrating HIV drug resistance data with molecular transmission network analysis, this study reveals several critical insights into the transmission and spread of drug-resistant HIV-1 strains in Baotou. Although the overall prevalence of PDR remains at a moderate level, the network structure shows preliminary signals of resistant-strain dissemination, particularly in mutations associated with NNRTIs and PIs (38). NNRTI-resistant mutations spread within molecular clusters, consistent with previous studies—mutations such as K103N have minimal impact on replicative fitness and can persist long-term in untreated individuals (39). These characteristics enable their silent transmission within sexually active networks. None of the drug-resistant nodes were identified as network hubs or high-value nodes, indicating that resistant strains have not yet proliferated via key transmitters. Research findings indicate that drug-resistant HIV-1 transmission in Baotou remains controllable, providing a critical window for public health interventions. Future epidemic control must maintain continuous surveillance of drug-resistant strains, as core individuals within transmission networks may accelerate the spread of resistant viruses across different populations. This underscores the need to integrate resistance monitoring with molecular network surveillance to enable early detection of transmissible resistance and targeted interventions.

This study has several limitations: incomplete sampling coverage may obscure some transmission chains, phylogenetic inference alone cannot definitively determine transmission directionality, the lack of INSTIs drug resistance data may limit clinical guidance value, and recall bias and reporting bias may exist during data collection.

## 5 Conclusion

This study confirms that older heterosexual men sustain active, localized HIV-1 transmission networks in Baotou. Targeted control measures addressing this high-risk subpopulation are imperative to supplement current MSM-centric prevention efforts. Moreover, the high prevalence of NNRTI resistance underscores the necessity for continuous molecular surveillance and universal pretreatment drug susceptibility testing to curb further HIV transmission and the spread of drug resistance.

## Data Availability

The original contributions presented in the study are publicly available. This data can be found here: [https://www.ncbi.nlm.nih.gov/genbank/PZ014999-PZ015361].

## Footnotes

1
https://www.chiplot.online/network_plot
