# Supplementary material for: Characterization of sexually acquired HIV-1 transmission networks and genetic variation in northern frontier China, 2021–2024
Source: Front Public Health. 2026 Jun 8;14:1791010. doi: 10.3389/fpubh.2026.1791010 (PMC13283965; doi:10.3389/fpubh.2026.1791010)
Supplement: Supplementary file 1 [file Supplementary_File_1.docx]

Supplementary Material

**1 Supplementary Figure 1. Transmission Network of HIV Drug-Resistant Strains in Baotou.**

#
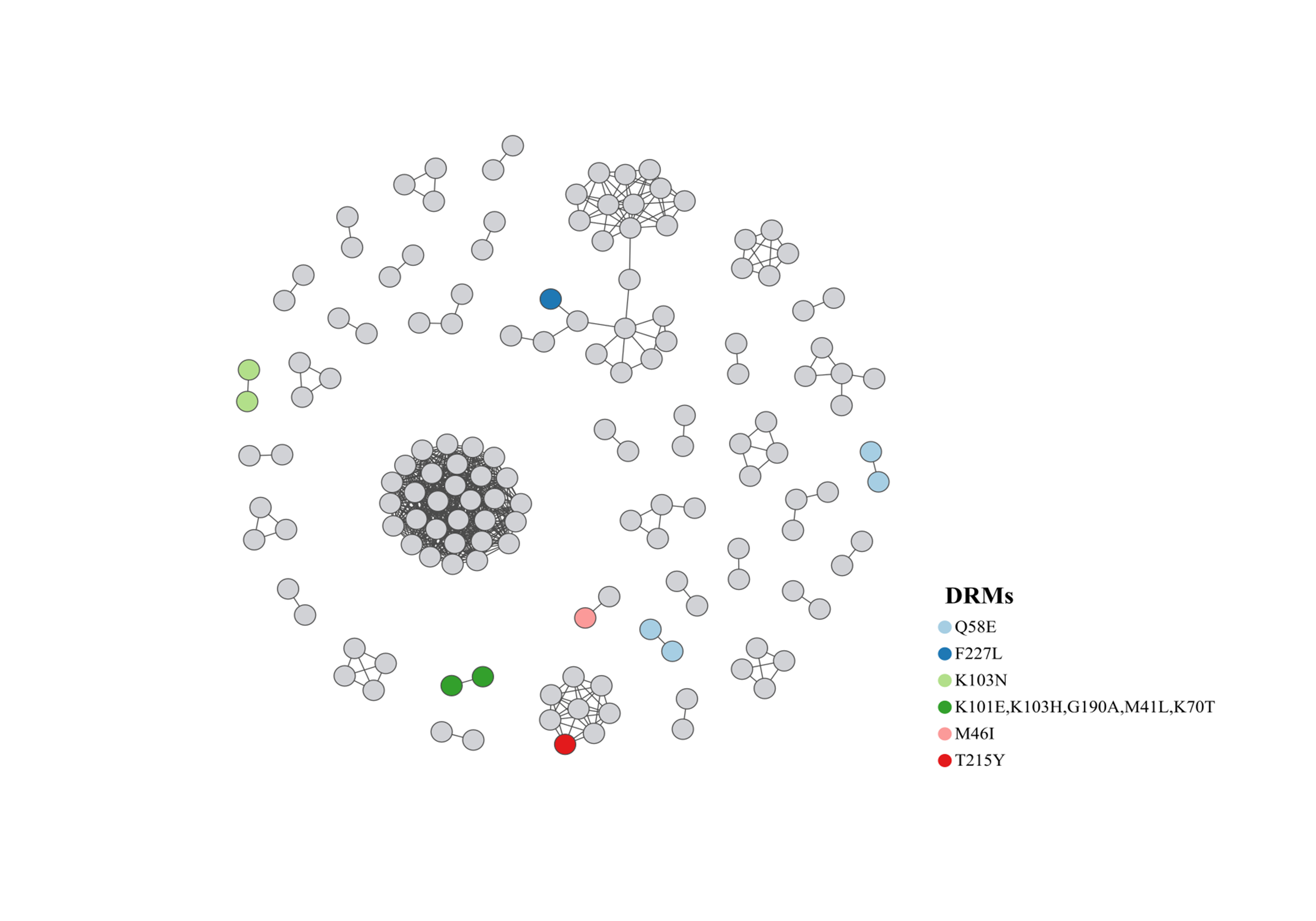


# 2 Supplementary Table 1. PCR primers.

|  | **Primer name** | **Sequence（5' to 3'）** | **Location on HXB2** |
| --- | --- | --- | --- |
| The first round of nested-PCR | DR-1 | TTGGAAATGTGCTAAGGAAGGAC | 2028-2050 |
|  | DR-2 | CACTCTCTGACCTGCTATCATCAT | 3529-3509 |
|  | DR-3 | CAGAGCGAACTGCCCCACCA | 2147-2166 |
|  | DR-4 | CTGCAATTTCTAGCTCTGCTTC | 3462-3441 |
| The second round of nested-PCR | *pol*-A_For | CAGAGCCAACATGCCCACCA | 2147-2166 |
|  | *pol*-A_Rev | GGGCCATCCGATCCTGGCTT | 2586-2605 |
|  | *pol*-B_For | GTTGACTAAGGTAGGTTGCAC | 2519-2539 |
|  | *pol*-B_Rev | CTGGTTCATTRTTKRTACTAGGT | 2945-2970 |
|  | *pol*-C_For | TTYTGGGARGTYCARYTAGTACC | 2808-2833 |
|  | *pol*-C_Rev | AGTTCATATGCCATCCAAAG | 3231-3250 |

# 3 Supplementary Table 2. Distribution of drug resistance mutations in 363 pol sequences.

|  | Number | PDR% | Drug Sensitivity^a^ | | |
| --- | --- | --- | --- | --- | --- |
|  |  |  | Low level | Intermediate | High level |
| **Total** | 29 | 7.99 |  |  |  |
| **PIs** | 8 | 2.20 |  |  |  |
| M46I | 2 | 0.55 |  | NFV |  |
| Q58E | 5 | 1.38 | TPV/r |  |  |
| N83D | 1 | 0.28 | NFV |  |  |
| **NRTIs** | 9 | 2.48 |  |  |  |
| L74LI | 1 | 0.28 | ABC |  | DDI |
| M41L,K70T | 2 | 0.55 | ABC\AZT\FTC\3TC\TDF | D4T\DDI |  |
| M184V | 1 | 0.28 | ABC |  | FTC\3TC |
| K65R | 1 | 0.28 | FTC\3TC | ABC\TDF | D4T\DDI |
| T215Y | 1 | 0.28 | DDI | D4T | AZT |
| D67E,K70N | 2 | 0.55 | ABC\AZT\DDI\TDF | D4T |  |
| D67del,S68del | 1 | 0.28 |  | ABC\AZT\FTC\3TC\TDF | D4T\DDI |
| **NNRTIs** | 16 | 4.41 |  |  |  |
| G190S | 1 | 0.28 | DPV\RPV |  | EFV\NVP |
| K101E | 1 | 0.28 | EFV | DPV |  |
| E138G | 2 | 0.55 | DPV\RPV |  |  |
| A98G | 1 | 0.28 | RPV | DPV\NVP |  |
| F227L | 1 | 0.28 | EFV | NVP | DOR |
| K103N,P225H | 1 | 0.28 |  | DOR | EFV\NVP |
| K103N | 5 | 1.38 |  |  | EFV\NVP |
| K101E,K103H,G190A | 2 | 0.55 | ETR |  | DPV\EFV\NVP\RPV |
| L100I | 2 | 0.55 |  | EFV\ETR\RPV | DPV\NVP |
